# Solution structure of the C-terminal domain of the measles virus V protein in its free form and mechanistic analysis of STAT2 targeting

**DOI:** 10.1128/jvi.00739-25

**Published:** 2025-09-11

**Authors:** Kaho Morita, Nanaka Goda, Madoka Kimoto, Satomi Inaba-Inoue, Nana Yabuno, Aoi Sugiyama, Hiroyuki Kumeta, Toyoyuki Ose

**Affiliations:** 1 Faculty of Advanced Life Science, Hokkaido University 12810, Sapporo, Japan; University Medical Center Freiburg, Freiburg, Germany

**Keywords:** STAT transcription factors, immune evasion, innate immunity, measles, paramyxovirus, protein–protein interactions, nuclear magnetic resonance, steady-state nuclear Overhauser effect

## Abstract

Viruses commonly evade the host antiviral interferon (IFN) response by targeting key components of the Janus kinase–signal transducer and activator of transcription (JAK–STAT) pathway, typically STAT1 and STAT2. Among the well-characterized viral IFN antagonists, measles virus (MeV), a member of the *Morbillivirus* genus, encodes a multifunctional V protein (MeV-V) that directly interacts with STAT proteins. The C-terminal domain (CTD) of MeV-V selectively binds to STAT2, disrupting the formation of the IFN-stimulated gene factor 3 (ISGF3) complex by inhibiting the STAT2–interferon regulatory factor 9 (IRF9) association. Here, we report a solution structure covering the MeV-V_CTD_ in its unbound form, as determined by nuclear magnetic resonance spectroscopy. While the overall architecture, including a distinctive zinc-finger motif, conforms to previously predicted features, our analysis reveals unexpected features, including distinct proline *cis* conformers that may have functional relevance. Molecular mapping analysis, combined with relaxation measurements, identified key residues implicated in STAT2 recognition and revealed substantial conformational flexibility within the domain. These findings suggest that MeV-V_CTD_ employs a shared binding surface for STAT2 binding as for melanoma differentiation-associated protein 5 (MDA5) interaction, underscoring its structural adaptability. As V proteins across *Morbillivirus* species engage diverse host pathways, including immune signaling, cell cycle regulation, and apoptosis, by targeting multiple proteins, we propose that the dynamic yet folded nature of the V_CTD_ underlies its ability to serve as a versatile interaction module in host–pathogen interplay.

**IMPORTANCE:**

The measles virus V protein, encoded by the P gene, orchestrates the broad modulation of host responses, including immune evasion, by interacting with multiple host factors. With regard to structural studies of V_CTD_, to date, only one protein from parainfluenza virus 5 has been crystallographically analyzed in complex with host targets. Despite the conserved nature of the V_CTD_ among paramyxoviruses, structural information on the unbound state of this domain is lacking, and current insights largely rely on computational predictions based on the structure of the bound form. Our nuclear magnetic resonance work provides the first structure of the V_CTD_ from paramyxoviruses in its free form. In accordance with our previously presented data, we further confirmed that the MeV-V binding site of STAT2 overlaps that of IRF9. The conformational flexibility observed within the folded CTD provides the structural basis for its ability to engage with multiple host targets with high specificity.

## INTRODUCTION

The Janus kinase–signal transducer and activator of transcription (JAK–STAT) signaling pathway, which comprises critical mediators of interferon (IFN) signaling, is frequently targeted by pathogenic viruses for immune evasion and modulation (1, 2). Interactions between viral factors and components of the JAK–STAT pathway exhibit remarkable diversity, ranging from antagonistic to stimulatory effects, and encompass both canonical (cytokine- and growth factor-driven) and non-canonical responses (1).

Among the viral strategies to counteract IFN systems, interference with STAT1 and STAT2, which are critical to the development of innate and adaptive immune responses, has been one of the most extensively studied.

Type I (primarily IFN-α/β) and type III (IFN-λ 1–4) IFNs promote phosphorylation of conserved tyrosine residues on STAT1 and STAT2, leading to the formation of a STAT1–STAT2 heterodimer. In association with interferon regulatory factor 9 (IRF9), the phosphorylated (pY) heterodimer (pY-STAT1–pY-STAT2) forms a heterotrimer called the IFN-stimulated gene factor 3 (ISGF3) complex. Upon nuclear import, ISGF3 binds to IFN-stimulated response elements (ISREs) within the genome. Type II IFN (IFN-γ) promotes the tyrosine phosphorylation of STAT1, leading to the formation of a pY-STAT1 homodimer (and/or N-terminal domain-tethered tetramer [3]) that binds to gamma-activated sequences (GAS) in DNA. ISRE and GAS elements collectively regulate the transcription of hundreds of IFN-stimulated genes (ISGs) and orchestrate antiviral responses (2).

Several viruses from the family *Paramyxoviridae* code for well-studied antagonists of the pY-STAT1–pY-STAT2 heterodimer (including ISGF3) system. Within this family, genera such as *Morbillivirus* (e.g., measles virus [MeV]), *Rubulavirus* (e.g., mumps virus), *Henipavirus* (e.g., Nipah virus and Hendra virus), *Respirovirus* (e.g., Sendai virus), and *Avulavirus* (e.g., Newcastle disease virus) encode proteins from the P gene, including P, V, W, D, I, and C proteins, which interact directly with STAT1 and/or STAT2.

The *Morbillivirus* genus comprises seven genetically closely related species, of which MeV remains a severe cause of global morbidity and mortality. Despite the availability of effective vaccines, MeV is responsible for up to 100,000 deaths annually (4). The MeV genome is a non-segmented, negative-sense RNA, approximately 16 kb in length. MeV infection induces profound immunosuppression, increasing susceptibility to secondary infections and mortality (5). In rare cases, MeV persists in the central nervous system for years following acute infection, leading to subacute sclerosing panencephalitis, a progressive neurological disorder (6, 7). The MeV P gene encodes three proteins: P, V, and C. The P protein, composed of 507 amino acid residues, functions as a cofactor for viral polymerase (L), while the C protein, generated from an alternative initiation codon, encodes a distinct 186-amino-acid protein (8). Furthermore, the mRNA editing of P (9), inserting a guanosine, produces a protein that shares its N-terminal 231 residues with MeV-P (the N-terminal region of MeV-P, [MeV-P_NT_] = the N-terminal region of MeV-V [MeV-V_NT_]), which is intrinsically disordered (10). However, the C-terminal domains (CTDs) of MeV-P and MeV-V differ completely. The MeV-V_CTD_ consists of 68 residues (residues 232–299) with a zinc-binding cysteine-rich domain (9). Among paramyxoviruses, the three-dimensional structure of V_CTD_ was experimentally determined using the V_CTD_ of parainfluenza virus 5 (PIV5). Two crystal structures of the PIV5 V_CTD_ were reported: one in complex with the ubiquitin ligase DDB1 and one with the helicase domain of melanoma differentiation-associated protein 5 (MDA5) (11, 12). Given the high sequence conservation (approximately 50% identity among paramyxoviruses), AlphaFold3 and other prediction tools suggest that MeV-V_CTD_ adopts the same characteristic zinc-finger fold with two zinc ions and a β-strand-based protrusion.

Previous studies have extensively characterized the interactions between MeV-V_NT_ and STAT1 (13–18), identifying amino acids 110–120 as the key region for STAT1 binding (15). In contrast, MeV-V_CTD_ has been shown to interact with STAT2; particularly, Trp240, Phe246, Asp248, and Trp250 are critical for STAT2 binding (14, 15, 19). Using purified STAT1, STAT2, and various constructs of MeV-V (intact, V_NT_, and V_CT151-299_ [residues 151–299]), we confirmed that MeV-V_NT_ selectively binds STAT1, whereas MeV-V_CTD_ specifically interacts with STAT2 (19).

In addition to direct interactions with STAT1/STAT2, MeV-V exhibits multifaceted functionality. It engages with several host factors involved in IFN production, including phosphatases PP1 (20), melanoma differentiation-associated protein 5 (MDA5) (21), laboratory of genetics and physiology 2 (LGP2) (22), nuclear factor-kappa B (NF-κB) subunit p65 (23), IκB kinase α (24), and IRF7 (24). MeV-V also modulates cell cycle and apoptosis pathways (binding to p53 and p73) (25, 26), interferes with the interleukin-1β secretion system (binding to NLRP3 inflammasome) (27), and disrupts mitochondrial function via interactions with 5′-aminolevulinate synthase 1 (ALAS1; binding to ALAS1) (28), thereby affecting mitochondrial dynamics.

Given that MeV-V_CTD_ is predicted to exhibit structural flexibility despite forming a defined three-dimensional structure, we performed structural and biophysical characterization using nuclear magnetic resonance (NMR). In this study, we determined the solution structure of MeV-V_CTD_ in its free form and revealed its similarity to PIV5-V_CTD_ in complex with its targets. Compared with the structure of the PIV5-V_CTD_ or computationally predicted MeV-V_CTD_ models, notable differences were observed in the conformations of the two proline peptide bonds as well as loop conformations. Steady-state nuclear Overhauser effect (NOE) analyses identified flexible regions within the MeV-V_CTD_. Furthermore, titration experiments of the STAT2-core provided plausible new binding sites within the MeV-V_CTD_. Competition assays further demonstrated that the STAT2 binding site for MeV-V_CTD_ overlaps that of the IRF9-IRF-associated domain (IRF9-IAD), which is consistent with a previous report (19). Taken together, our findings characterize the well-conserved paramyxovirus V_CTD_ in its free form and highlight its intrinsic flexibility, which likely contributes to its functional versatility.

## RESULTS AND DISCUSSION

### Preparation of MeV-V_CT221-299_ and interaction analysis using isothermal titration calorimetry (ITC)

**Fig 1:**
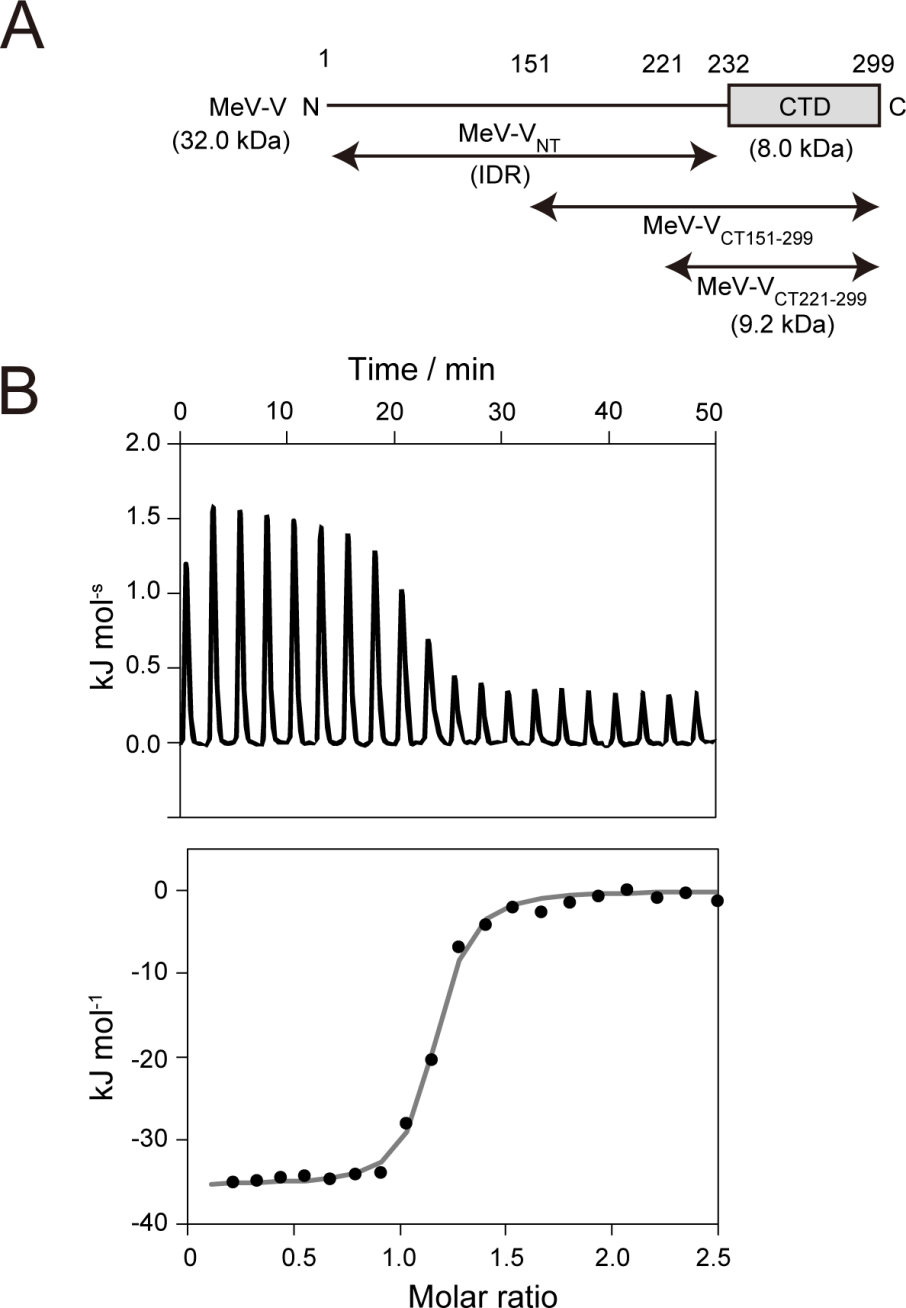
(**A**) Schematic representation of the domain organization of the V protein. The N-terminal region (V_NT_) contains an intrinsically disordered region (IDR). The construct used in this study (MeV-V_CT221-299_) contains the CTD. (**B**) ITC analysis of MeV-V_CT221-299_ binding to STAT2-core. The upper panel shows a representative titration thermogram after blank value subtraction (exothermic peaks are oriented upward). The lower panel displays the integrated heat data fitted using a 1:1 binding model.

Previously, we quantified the binding affinities of MeV-V (full-length) with STAT2, MeV-V with STAT2-core, and MeV-V_CT151-299_ with STAT2-core, demonstrating that MeV-V_CT151_ is sufficient for STAT2 targeting (19). In this study, we designed a shorter MeV-V_CT_ construct, termed MeV-V_CT221-299_ (residues 221–299), for NMR spectroscopy (Fig. 1A). The elution profile of size-exclusion chromatography (SEC), which is the last step in the purification process, and the SDS-PAGE demonstrated that the purity of MeV-V_CT221-299_ was sufficient for further analysis (Fig. S1A through C). Purified MeV-V_CT221-299_ was subjected to interaction analysis with STAT2-core using ITC. From the titration experiment of MeV-V_CT221-299_ into STAT2-core, typical exothermic signals were observed over the injection of MeV-V_CT221-299_ (Fig. 1B, top). This titration profile was well fit with a 1:1 binding model (Fig. 1B, bottom), enabling the determination of thermodynamic parameters from three independent experiments. The dissociation constant (*K*_D_) for the interaction was determined to be 0.212 ± 0.085 µM at 20°C, which is comparable to that between MeV-V and STAT2-core (0.18 ± 0.1 µM) at 10°C (19). This interaction is strongly enthalpy-driven and follows a 1:1 stoichiometry, indicating that one MeV-V_CT221-299_ molecule interacts with one STAT2-core molecule (Table 1). As shown in Fig. S1A and B, MeV-V_CT221-299_ exists as a monomer, further supporting this binding model. These results define the minimal MeV-V region required for STAT2 targeting. Consequently, MeV-V_CT221-299_ was used in all the NMR experiments conducted in this study.

**TABLE 1:** Thermodynamic parameters for the interaction between MeV-V_CT221-299_ and STAT2-core quantified by ITC

Parameter	MeV-V_CT221-299_ and STAT2
*K*_D_ (M)	(2.12 ± 0.85) ×10^−7^
*n* (stoichiometry)	1.17 ± 0.05
Δ*G* (kJ/mol)	−37.6 ± 0.9
Δ*H* (kJ/mol)	−52.3 ± 4.5
−*T*Δ*S* (kJ/mol)	14.7 ± 4.9

### Characterization of MeV-V_CT221-299_ by NMR

**Fig 2:**
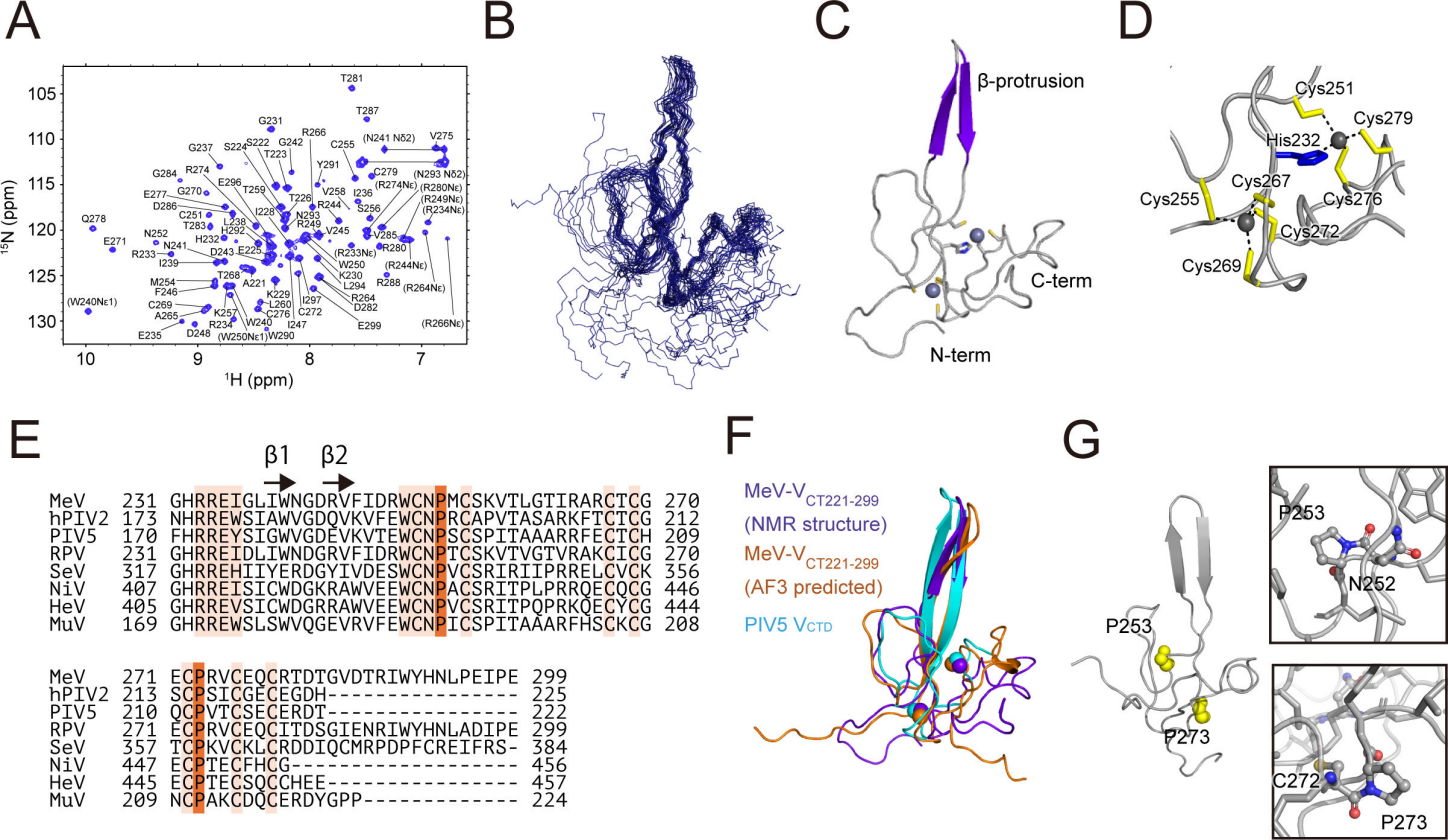
NMR characterization of MeV-V_CT221-299_. (**A**) Assigned two-dimensional ^1^H–^15^N HMQC spectrum of ^15^N-MeV-V_CT221-299_. The assigned peaks are labeled. Side-chain resonances of Arg (Hε/Nε, aliased), Trp (Hε1/Nε1), and Asn (Hδ2s/Nδ2) are indicated in parentheses. (**B**) Ensemble of the 20 lowest-energy NMR structures of MeV-V_CT221-299_ with Cα traces shown. (**C**) Ribbon diagram of the lowest-energy structure. (**D**) Coordination geometry of the two zinc ions. The backbone is shown in gray, cysteine side chains in yellow, and the coordinating histidine side chain in blue. (**E**) Multiple sequence alignment of the CTD from paramyxovirus V proteins: MeV (strain Ichinose-B95a; UniProt, P0C774), human parainfluenza virus 2 (hPIV2, P19847), PIV5 (P11207), rinderpest virus (RDV, Q03340), Sendai virus (SeV, P69282), Nipah virus (NiV, Q997F2), Hendra virus (HeV, O55777), and mumps virus (MuV, P33483). Fully conserved residues are highlighted; two prolines found in the *cis* conformation in the free MeV-V_CTD_ structure are highlighted in orange. The secondary structure (analyzed by DSSP [29]; two β-strands) of MeV-V_CT221-299_ is indicated by arrows. (**F**) Structural comparison of the solution structure of unbound MeV-V_CT221-299_ (purple), AlphaFold3-predicted model (orange), and the crystal structure of PIV5-V_CTD_ from the crystal in complex with MDA5 (cyan). Zinc ions are represented as spheres. (**G**) Location of the two *cis*-proline residues. Insets show zoomed-in views highlighting the *cis* peptide bonds.

NMR spectroscopy was used to investigate the structural properties of MeV-V_CT221-299_ in solution. For signal assignment, we prepared ^13^C-, ^15^N-labeled MeV-V_CT221-299_ and assigned the ^15^N, NH, ^13^Cα, ^13^Cβ, and C´ resonances using triple-resonance experiments. Out of the 74 residues, the backbone amide resonances of 70 residues were assigned in the ^1^H–^15^N heteronuclear multiple quantum correlation (HMQC) spectrum, except for Thr262, Ile263, Cys267, and Ile289 (Fig. 2A). The chemical shift data were further analyzed to assess the secondary structure and loop regions using secondary structure propensity (SSP) and the random coil index (RCI) (Fig. S1). A calculated SSP score at a specific residue of 1 or −1 indicates fully formed α- or β-structure, respectively (29). SSP analysis of MeV-V_CT221-299_ revealed that residues 231–239 and 245–251 exhibited scores ranging from –1.0 to –0.5, indicative of β-strand formation. Additionally, residues 263–275 displayed SSP scores between –0.6 and 0.1, suggesting a partially β-stranded conformation. An RCI score at a specific residue reflects protein flexibility calculated from NMR chemical shifts (30). RCI analysis of MeV-V_CT221-299_ identified residues 221–229 and 293–299 as regions of increased structural flexibility, with scores exceeding 0.1. These findings provide insights into the conformational dynamics of MeV-V_CT221-299_, highlighting the regions with ordered secondary structures and flexible segments.

### Solution structure calculation of MeV-V_CT221-299_

The solution structure of MeV-V_CT221-299_ was determined using the NOE-derived distance restraints and backbone dihedral angles (Table 2). Previous studies have demonstrated that MeV-V and MeV-V_CT151-299_ exist as monomers in solution, as assessed using SEC coupled with multi-angle light scattering (19). The elution profile of MeV-V_CT221-299_ from SEC also demonstrated a monomeric state: calculated molecular mass from the standard curve is 5.6 kDa (theoretical mass, 9. 2kDa) (Fig. S1).

**TABLE 2:** CYANA structure calculation statistics

Statistics of NMR analysis	Result
Upper distance limits	
Total	869
Short range (|*i*-*j*| ≤ 1)	603
Medium range (1 < |*i*-*j*| < 5)	109
Long range (|*i*-*j*| ≥ 5)	157
Dihedral angle limits	
Total	69
phi	35
psi	34
Target function value	0.64
Violations	
Distance > 0.2 Å	8
>0.1 Å	27
Angle > 1°	4
RMSD^*a*^ (residue range 231–279)	
Backbone atoms	0.77
Heavy atoms	1.23
Ramachandran plot	
Most favored	65.6%
Additionally allowed	31.9%
Generously allowed	2.2%
Disallowed	0.2%

^*a*^ RMSD, root mean square deviation.

Figure 2B shows the superposition of the final 20 energy-minimized structures of MeV-V_CT221–299_. The root mean square deviation for the structured region (residues 231–279) was 0.77 Å for the backbone atoms and 1.26 Å for all heavy atoms. The Ramachandran analysis indicated that 65.6% of the residues reside in the most favored regions, 31.9% in the additionally allowed regions, 2.2% in the generously allowed regions, and 0.2% in the disallowed regions. The core structural features of MeV-V_CTD_ were expected to include a distinctive zinc-finger motif, as demonstrated by the crystal structure of the PIV5-V_CTD_ and X-ray absorption studies of MeV-V (11, 12, 19). Therefore, for structural refinement, two zinc ions were introduced, coordinated by one histidine (residue 232) and seven cysteine residues (residues 251, 255, 267, 269, 272, 276, and 279) (Fig. 2C and D), all of which are conserved among paramyxovirus V proteins (31) (Fig. 2E). The final structure adopts a characteristic zinc-finger fold with two zinc ions and a β-strand-based protrusion (residues 235–250) consisting of two β-strands. Apart from the two β-strands, the majority of the structure is composed of long, flexible loops. The overall fold resembles that of PIV5-V_CTD_ in complex with MDA5 (11), PIV5-V in complex with DDB1 (12), or the AlphaFold-predicted structure of MeV-V_CT221-299_ (Fig. 2F). However, in this study, structural flexibility was directly observed via the steady-state ^1^H–^15^N NOE (ssNOE) measurements using NMR (see below).

Using the chemical shift difference between ^13^C_β_ and ^13^Cγ, the information on the Xaa-Pro peptide bond conformation can be obtained in a reference-independent manner (Xaa, any amino acid residue) (32). Interestingly, chemical shift differences between ^13^C_β_ and ^13^C_γ_ of two proline residues clarified that Pro253 and Pro273 predominantly adopt the *cis* conformation in the free form of MeV-V_CT221-299_ (C_β_, 34.65 ppm; C_γ_, 24.74 ppm; difference, 9.91 for Pro253; and C_β_, 34.54 ppm; C_γ_, 24.77 ppm; difference, 9.77 for Pro273) (Fig. 2G). No exchange reactions were observed with the *trans* form for these prolines. In contrast, the remaining three proline (residues 227, 295, and 298; not conserved) residues were assigned to the *trans* conformation. These two proline residues (Pro253 and Pro273) are conserved among the V proteins from paramyxoviruses (Fig. 2E). While the conformational states of Pro253 and Pro273 in the STAT2-bound form of MeV-V_CTD_ remain undetermined, it is noteworthy that the corresponding residues in PIV5-V (Pro192 and Pro212) adopt *trans* conformations in the crystal structures when bound to MDA5 and DDB1 (11, 12). Although further experimental validation is required, the observed two *cis* conformations in free MeV-V_CT221-299_ may facilitate a favorable binding reaction, potentially transitioning to the *trans* conformation upon interaction with target molecules such as STAT2.

### Titration of STAT2-core on MeV-V_CT221-299_ monitored by NMR

**Fig 3:**
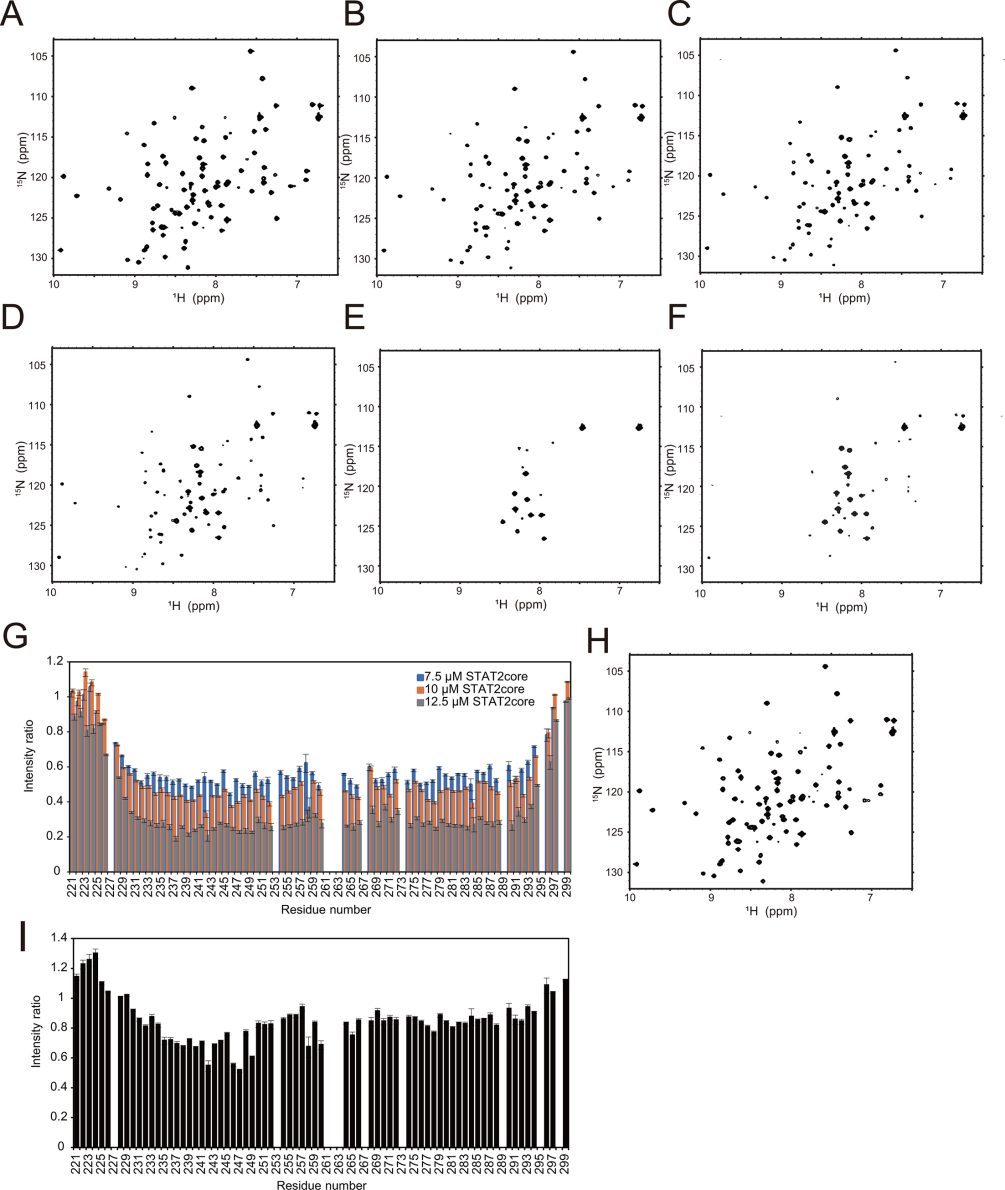
Two-dimensional NMR characterization of MeV-V_CT221-299_. (A–E) ^1^H–^15^N HMQC spectra of ^15^N-MeV-V_CT221-299_ (20 µM) in the absence of STAT2-core (**A**), and in the presence of increasing concentrations of STAT2-core: 7.5 µM (**B**), 10 µM (**C**), 12.5 µM (**D**), and 20 µM (**E**). (**F**) ^1^H–^15^N HMQC spectrum of ^15^N-MeV-V_CT221-299_ (20 µM) in the presence of STAT2-core (20 µM) and IRF9-IAD (20 µM). (**G**) Residue-specific changes in signal intensities in the ^1^H–^15^N HMQC spectrum of ^15^N-MeV-V_CT221-299_ upon STAT2-core titration. Relative intensities are plotted for each residue in the presence of 7.5 µM (blue), 10 µM (orange), and 12.5 µM (gray) STAT2-core. Error bars, calculated from estimated noise values of two spectra, are shown. (**H**) ^1^H–^15^N HMQC spectra of ^15^N-MeV-V_CT221-299_ (20 µM) in the presence of IRF9-IAD (20 µM). (**I**) Residue-specific signal intensity changes in ^15^N-MeV-V_CT221-299_ (20 µM) upon addition of IRF9-IAD (20 µM) alone.

To investigate the interaction between MeV-V_CT221-299_ and STAT2-core, we performed NMR titrations monitored by band-selective optimized flip-angle short-transient (SOFAST) ^1^H–^15^N HMQC spectroscopy (33). Owing to the large increase in the molecular weight upon binding (9.2 kDa [MeV-V_CT221-299_ ] to 74.9 kDa [MeV-V_CT221-299_ and STAT2-core]), a reduction in the intensity of the NMR signals around the binding site of MeV-V_CT221-299_ in the ^1^H–^15^N HMQC spectrum, instead of changes in each chemical shift, was expected. ^15^N-labeled MeV-V_CT221-299_ was titrated with STAT2-core at molecular ratios of 1:0.375, 1:0.5, 1:0.64, and 1:1, resulting in a progressive reduction in the resonance intensities for MeV-V_CT221-299_ (Fig. 3A through E and G). Although these data do not precisely define the binding interface, they confirm that MeV-V_CT221-299_ interacts with STAT2-core, consistent with the ITC results. Notably, the signal intensities of the N-terminal (residues 221–228) and C-terminal (residues 294–299) regions remained largely unchanged relative to those of the other regions, suggesting that these terminal segments are not directly involved in the interaction.

### Effect of the presence of IRF9-IAD

The reduced signal intensity observed in the ^1^H–^15^N HMQC spectrum of ^15^N-MeV-V_CT221-299_ upon STAT2-core binding was slightly restored when an equimolar amount of IRF9-IAD was introduced (Fig. 3F). This indicates an increase in the unbound fraction of ^15^N-MeV-V_CT221-299_. This can be explained by a certain fraction of STAT2-core being unable to interact with ^15^N-MeV-V_CT221-299_ because that fraction of STAT2-core binds to added IRF9-IAD; this phenomenon happens if the same binding surface of STAT2-core for the two molecules (MeV-V_CT221-299_ and IRF9-IAD) is used. Previously, surface plasmon resonance and SEC analyses suggested that the STAT2 binding site for MeV-V overlaps that for IRF9-IAD (19). Our new NMR results further confirmed that MeV-V competes with IRF9-IAD for STAT2 binding.

Additionally, the titration of ^15^N-MeV-V_CT221-299_ with IRF9-IAD alone resulted in reduced signal intensity in the ^1^H–^15^N HMQC spectrum of ^15^N-MeV-V_CT221-299_ (Fig. 3H and I), suggesting a weak interaction between the two proteins resulting from the emergence of ^15^N-MeV-V_CT221-299_ in complex with IRF9-IAD. This weak interaction was not able to be observed in the previous ITC experiment (19).

Notably, when all three proteins—MeV-V_CT221-299_, STAT2-core, and IRF9-IAD—were present in equimolar amounts, the signal intensity of MeV-V_CT221-299_ was restored, suggesting that the STAT2–IRF9-IAD interaction is stronger than the MeV-V_CT221-299_–IRF9-IAD interaction. The weak interaction between MeV-V and IRF9-IAD may influence the competition between MeV-V and IRF9 for STAT2 binding, potentially affecting the regulatory mechanisms underlying STAT2 targeting.

### Binding surface is highlighted using the ssNOE experiment

**Fig 4:**
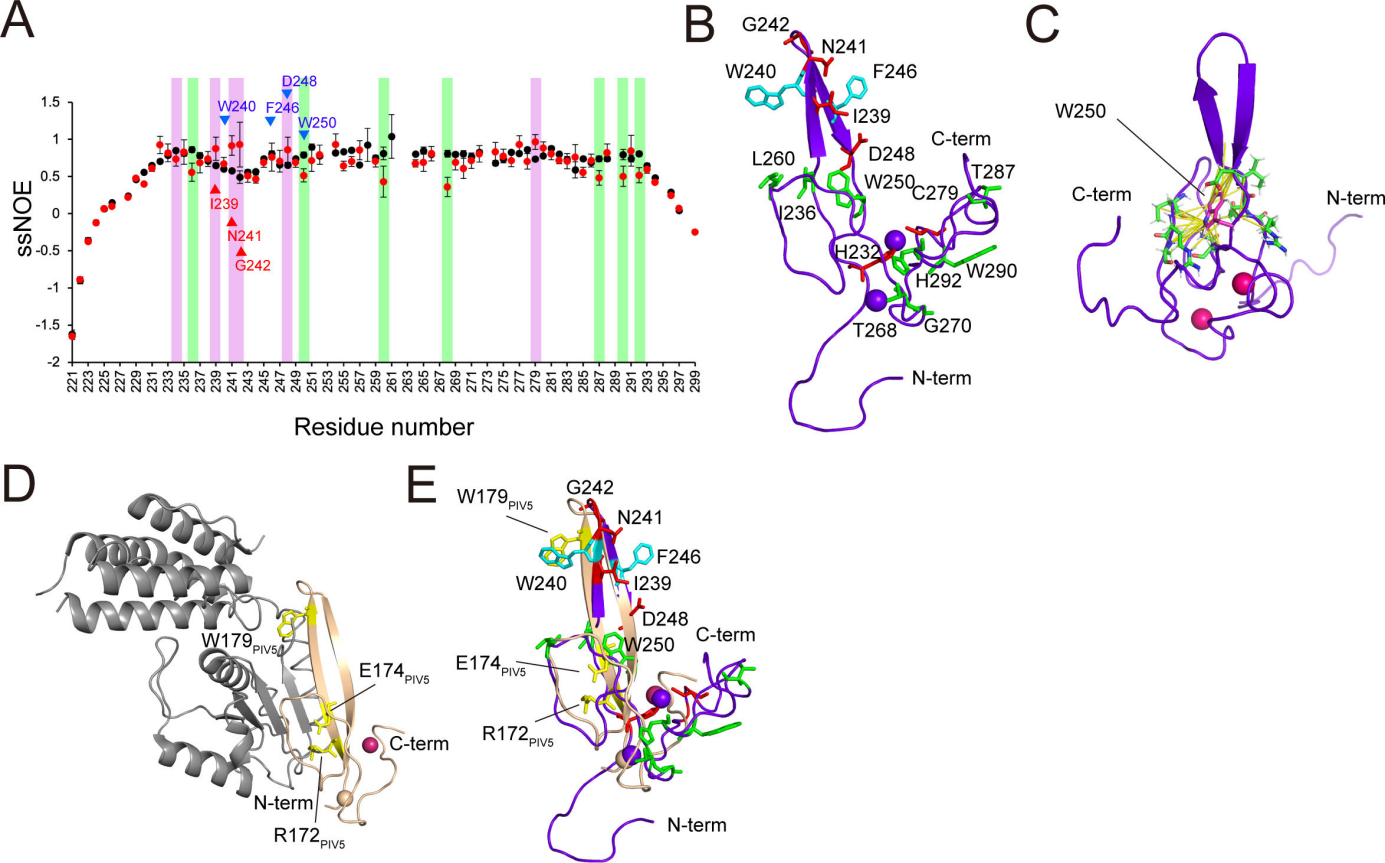
Mapping of the STAT2-core binding interface on MeV-V_CT221-299_ using ^15^N-^1^H NOE measurements and structural comparison. (**A**) Comparison of the ^1^H–^15^N ssNOE values for MeV-V_CT221-299_ in the absence (black) and presence (red) of STAT2-core. Error bars, calculated from estimated noise values of two spectra at each residue, are shown. Residues showing a NOE increase >0.2 upon STAT2 binding (His232, Ile239, Asn241, Gly242, Asp248, and Cys279) are highlighted in pink. Residues with a NOE decrease >0.2 (Ile236, Trp250, Leu260, Thr268, Thr287, Trp290, and His292) are highlighted in green. Residues previously reported as critical for STAT2 interaction are marked with blue triangles. Newly identified residues as candidates to interact with STAT2-core are marked with red triangles. (**B**) Cα-traced ribbon model of MeV-V_CT221-299_ showing residues identified from the NOE data in (**A**). Residues with increased NOE values are shown in red; those with decreased values are shown in green. Additional residues known to be important for STAT2 interaction (Trp240, Phe246) are labeled in cyan. (**C**) Trp250 located at the bottom of the β-protrusion is shown in magenta. The 33 NOEs observed in middle and long range (1 < |*i-j*|, where *i*, *j* are residue numbers) involving the Trp250 side chain are depicted in yellow. Interacting residues are shown as green stick models. (**D**) Crystal structure of PIV5-V_CTD_ in complex with MDA5, with PIV5-V_CTD_ in light brown and MDA5 in gray. Side chains involved in binding are shown in yellow. (**E**) Superimposed structures of MeV-V_CT221_ (purple) and PIV5-V_CTD_ (wheat). STAT2-interacting residues in MeV-V_CT221-299_, previously known (cyan) and newly suggested from ssNOE analysis (red), are labeled. Important residues of PIV5-V_CTD_ for the side-chain-specific interaction are indicated (yellow) and labeled as in (**D**).

To obtain insights into the changes in protein dynamics between the free and STAT2-core-bound forms of MeV-V_CT221-299_, the ssNOE measurements were performed. The ssNOE values of ^15^N-MeV-V_CT221-299_, both in the absence and the presence of STAT2-core, are shown in Fig. 4A. In the absence of STAT2-core, MeV-V_CT221-299_ exhibited low ssNOE values across the molecule, indicative of a highly dynamic and flexible structure. When mixed with STAT2-core, residues His232, Ile239, Asn241, Gly242, Asp248, and Cys279 displayed increased ssNOE values (more than 0.2 beyond the error threshold), suggesting localized rigidification upon interaction. Together with the ITC result that the interaction between MeV-V_CT221-299_ and STAT2-core is entropy-unfavorable, this suggests that the complex formation includes structural rigidification upon binding, resulting in a loss of conformational flexibility.

Previous experiments using a yeast two-hybrid system and reporter gene assay revealed that the minimum region of MeV-V to impair the IFN-α/β signaling is MeV-V_232-280_ (15). In particular, Trp240, Phe246, Asp248, and Trp250 were shown to be critical for STAT2 interactions (14, 15, 19). Our ssNOE study extended this understanding by revealing additional residues (Ile239, Asn241, and Gly242) that likely contribute to an expanded binding interface with STAT2-core (Fig. 4B). The ssNOE values of His232 and Cys279 also increased in the presence of STAT2-core; the side chains of these two residues are used to coordinate one of the zinc ions, indicating that they do not directly interact with STAT2-core.

In contrast, when mixed with STAT2-core, residues Ile236, Trp250, Leu260, Thr268, Thr287, Trp290, and His292 displayed decreased ssNOE values (by more than 0.2 beyond the error threshold), suggesting obtained flexibility upon STAT2-core binding (Fig. 4A). Among them, the Trp250 was of interest because we previously reported that purified MeV-V (W250A) completely lost its binding activity to STAT2 (19). If Trp250 directly interacts with STAT2-core, the value of ssNOE should increase in the presence of STAT2-core. The side chain of Trp250, located in the base of the β-protrusion, was observed to have as many as 33 NOEs with middle and long range (1 < |*i-j*|, where *i*, *j* are residue numbers), stabilizing the β-protrusion rather than being exposed to the solvent (Fig. 4C). The corresponding residue of PIV5-V_CTD_, Trp189, plays the same role in the complex structure with MDA5 and DDB1 (11, 12) not providing direct interaction with the target molecules. Therefore, rather than being directly used for the interaction, Trp250 in MeV-V plausibly contributes to keeping the structure of the β-protrusion, which was previously identified to be a major factor for the interaction with STAT2.

In the crystal structure of human parainfluenza virus 5 (hPIV5)-V_CTD_ in complex with MDA5, one side of β1 within the β-protrusion forms an extended β-sheet with the β-sheet of the MDA5 domain 2A, while the opposite side interacts with β2 of hPIV5-V_CTD_ to further stabilize the β-sheet (Fig. 4D). The β-sheet-mediated interaction with MDA5, along with side-chain-specific contributions from hPIV5-V_CTD_ using Arg172, Glu174, and Trp179, supports a conserved binding mode among paramyxovirus V proteins (11), given the sequence homology (Fig. 2E). Therefore, a similar interaction between MeV-V_CTD_ and MDA5 was also expected (11). Figure 4E compares the free-state structure of MeV-V_CT221-299_ (this study) with that of hPIV5-V_CTD_, indicating important residues for the interaction with each target. In the structure of MeV-V_CT221-299_, we suggest that critical residues for the direct interaction are spread on the β-protrusion, and the binding site for STAT2 overlaps that for MDA5.

As IRF9-IAD binds to STAT2 at its coiled-coil domain (CCD) (34), and MeV-V competes with IRF9-IAD for the same binding site, MeV-V_CT221-299_ can also be predicted to target the CCD of STAT2. Our ssNOE data suggested that the MeV-V_CT221-299_ utilizes a similar interface to recognize STAT2 and MDA5 (Fig. 4E). Notably, paramyxovirus V_CTD_ are known to engage multiple host targets to facilitate immune evasion. Although these domains adopt a distinct three-dimensional fold, their inherent flexibility may enable recognition of diverse target molecules using the same binding sites, similar to intrinsically disordered proteins, which undergo structural adaptation upon binding to different partners (35). A deeper understanding of the molecular basis of MeV-V targeting, such as that on STAT2 and MDA5, might facilitate the development of new antiviral strategies including MeV-V inhibitors or safer vaccine strains.

## MATERIALS AND METHODS

### Construction of the expression plasmid of recombinant MeV-V_CT221-299_

To generate the MeV-V_CT221-299_ expression vector, the human rhinovirus 3C protease (HRV-3C) cleavage site was introduced between Arg220 and Ala221 of MeV-V_CT151-299_ (19) (in pGEX-6P-2 vector, GE Healthcare, USA) by PCR using primers of 5′-TTTCAGGGCCCGGCCAGCACTTCCGAGACACC-3′ and 5′-CAGCACTTCCAGCCTACTGGGGTTCGGGGG-3′. A pre-existing HRV-3C cleavage site after GST-tag was subsequently removed by PCR using primers of 5′- CCCCTGGGATCCCCAGG-3′ and 5′- AACTTCCAGATCCGATTTTGGAGG-3′. The PCR products were sequenced using an Applied Biosystems 3130 Genetic Analyzer (Life Technologies, Carlsbad, USA). The resulting MeV-V_CT221-299_ after the 3C protease digestion was designed to contain additional Gly and Pro before Ala221.

### Expression and purification of recombinant proteins

For the expression of MeV-V_CT221-299_, *Escherichia coli* strain BL21 star (DE3) (Thermo Fisher Scientific, USA) was transformed using the expression vector. A single colony of the transformant was inoculated into 5 mL of Luria-Bertani (LB) medium (with 100 µg/mL of ampicillin) and incubated at 37°C and 150 rpm with shaking. Then, 5 mL of the pre-culture was inoculated into 1 L of LB medium in 2 L of baffled flasks and shaken at 150 rpm and 37°C. Isopropyl β-D-thiogalactopyranoside (IPTG, 1 mM final concentration) was added when the culture reached an optical density at 600 nm (OD600) of 0.6, followed by incubation at 16°C for 16 h. Finally, cells were harvested by centrifugation.

The cells were resuspended to 0.2 mg/mL (final concentration) of cell lysis buffer A [phosphate-buffered saline (PBS), pH 7.3, 1 mM dithiothreitol (DTT), DNase I (Merck, USA), hen-egg lysozyme (Wako, Japan)] and disrupted using a sonicator. The cell solution was centrifuged at 10°C and 40,000 × *g* for 30 min, and the supernatant was collected. After filtration, Glutathione Sepharose 4B (Cytiva, USA) was added into the soluble fraction, which was resuspended overnight. After washing with buffer A, MeV-V_CT221-299_ was eluted from the resin using His-tagged 3C protease in the presence of 5 mM DTT. The elution was passed through a HisTrap HP column (Cytiva) to remove the protease. The proteins were further purified via hydrophobic interaction chromatography (HIC) using a HiTrap Butyl FF column (Cytiva) with HIC binding buffer [10 mM HEPES, pH 6.8, 150 mM NaCl, 1.2 M ammonium sulfate, 1 mM Tris (2-carboxyethyl) phosphine (TCEP)] and HIC elution buffer (10 mM HEPES, pH 6.8, 1 mM TCEP). Expression and purification of the unlabeled STAT2-core and IRF9-IAD have been described elsewhere (19).

### ITC

ITC experiments were performed using a NanoITC (TA Instruments, USA) at 20°C. Prior to the measurements, all solutions were degassed to remove air bubbles. The titrations consisted of 20 sequential injections of 2.5 µL of 250 µM MeV-V_CT221-299_ into a sample cell containing 35 µM STAT2-core in 10 mM HEPES (pH 7.4), 150 mM NaCl, and 1 mM TCEP. The injection interval was set to 150 s, and the stirring speed was 200 rpm.

The data were analyzed using NanoAnalyze software provided by the manufacturer and fitted to a 1:1 binding model to determine the dissociation constant (*K*_D_), enthalpy change (Δ*H*), and stoichiometry (*n*). The Gibbs free energy change (Δ*G*) and the entropy change (Δ*S*) were derived from the following equations:


$$\Delta G=RT\;\ln\;K_{D},$$

ΔG=ΔH−TΔS,

where *R* is the molar gas constant.

The thermodynamic parameters are summarized in Table 1, with errors representing the standard deviation of three independent measurements.

### Protein isotope labeling for NMR studies

Isotopically labeled MeV-V_CT221-299_ for NMR studies was prepared by growing bacterial cells (BL21 star [DE3]) in minimal M9 medium. ^13^C-, ^15^N-labeled samples were prepared for the backbone and side-chain assignment of MeV-V_CT221-299_ by supplementing the growth medium with ^15^NH_4_Cl (1 g L^−1^) and ^13^C_6_ glucose (5 g L^−1^). The ^15^N-labeled samples were prepared by supplementing the growing medium with ^15^NH_4_Cl (1 g L^−1^). Cells were typically harvested at OD600 = 0.8, and protein expression was induced with IPTG (0.2 mM final concentration). Purification was conducted as described for unlabeled MeV-V_CT221-299_. After HIC, the protein fractions underwent dialysis with the dialysis buffer (10 mM HEPES, pH 6.8, 1 mM TCEP) and were concentrated using a 3 kDa molecular weight cutoff (MWCO) Amicon centrifugal concentrator (Millipore, USA). 2-Methyl-2,4-pentandiol (MPD) with the final concentration of 5% was added before concentration.

### NMR spectroscopy

NMR experiments were performed using a Bruker Avance NEO 800 MHz spectrometer equipped with a TCI CryoProbe (CPTCI). NMR spectra were typically recorded with sample concentration of 130 µM in 10 mM HEPES (pH 6.8), 1 mM TCEP, 5% MPD, and 3.8% D_2_O at 20°C. The two-dimensional [^1^H–^15^N] HMQC spectra were measured using the SOFAST method (33). HN-detected 3D spectra, except for ^15^N-edited nuclear Overhauser effect spectroscopy (NOESY)–HSQC, were recorded using 20%–25% non-uniform sampling. The spectra were reconstructed using a compressed sensing algorithm (36). Data were processed using TopSpin-4.1.4 (Bruker Co., Ltd.) and NMRPipe 4.1 (37), and analyzed using Sparky 3.113 (https://www.cgl.ucsf.edu/home/sparky/manual/indx.html). The ^1^H-, ^13^C-, and ^15^N resonances were assigned using the following set of spectra: [^1^H–^15^N] HMQC, [^1^H–^13^C] HSQC, HN(CO)CA, HNCA, CBCA(CO)NH, HNCACB, C(CO)NH, HCCH–TOCSY, ^13^C-edited NOESY–HSQC, and ^15^N-edited NOESY–HSQC (mixing time 75 ms). All chemical shifts were referenced to 4,4-dimethyl-4-silapentane-1-sulfonic acid according to the IUPAC recommendations: (^15^N/^1^H) = 0.101329118 and (^13^C/^1^H) = 0.251449519.

For ssNOE measurement, 120 µM of ^15^N-MeV-V_CT221-299_ was prepared in 10 mM HEPES (pH 6.8), 75 mM NaCl, 1 mM TCEP, 2.5% MPD, and 3.8% D_2_O (20°C). For comparison, 24 µM of STAT2-core was further added under the same conditions. The error values of the ssNOE experiments were calculated using the following equation:


$$Error_{ssNOE}=(I_{1}/I_{2})\{{(S/N{)}_{1}}^{-2}+{(S/N{)}_{2}}^{-2}\}.$$

For the ^1^H–^15^N HMQC titration experiments, samples without labeling were mixed with 20 µM ^15^N labeled MeV-V_CT221-299_.

The prediction of secondary-structured and random regions was performed using SSP (29) and RCI (30).

### Structure calculation

Distance restraints were derived from the inter-proton NOE. Restraints on the backbone phi and psi torsion angles were derived from the chemical shifts of the backbone atoms using the TALOS program (38). The structure was calculated using the CYANA 2.1 software package (39). A total of 869 distance and 69 angle restraints were used for the final structure calculation. A total of 200 structures were calculated, and their quality was checked using PROCHECK-NMR (40) for the 20 lowest-energy structures. Finally, the ensemble of the 20 lowest-energy structures was determined. Structures were visualized using PyMOL 1.7.4 (http://www.pymol.org/).

## Data Availability

Structural coordinates and NMR resonance assignments for MeV-V_CT221-299_ were deposited in the BioMagResBank (BMRB; entry 53023 [https://bmrb.io/data_library/summary/?bmrbId=53023]) and Protein Data Bank (PDB; entry 9UJO), respectively.

## SUPPLEMENTAL MATERIAL

Fig. S1 jvi.00739-25-s0001.tif Biochemical characterization of MeV-V_CT221-299_.

Supplemental legends jvi.00739-25-s0002.docx Legends for Fig. S1 and Videos S1 to S3.

Video S1 jvi.00739-25-s0003.mov Rotation along the y axis of Fig. 2B.

Video S2 jvi.00739-25-s0004.mov Rotation along the y axis of Fig. 4B.

Video S3 jvi.00739-25-s0005.mov Rotation along the y axis of Fig. 4D.

ASM does not own the copyrights to Supplemental Material that may be linked to, or accessed through, an article. The authors have granted ASM a non-exclusive, world-wide license to publish the Supplemental Material files. Please contact the corresponding author directly for reuse.
