# Supplementary figures and images for: Solution structure of the C-terminal domain of the measles virus V protein in its free form and mechanistic analysis of STAT2 targeting
Source: J Virol. 2025 Sep 11;99(10):e00739-25. doi: 10.1128/jvi.00739-25 (PMC12548473; doi:10.1128/jvi.00739-25)

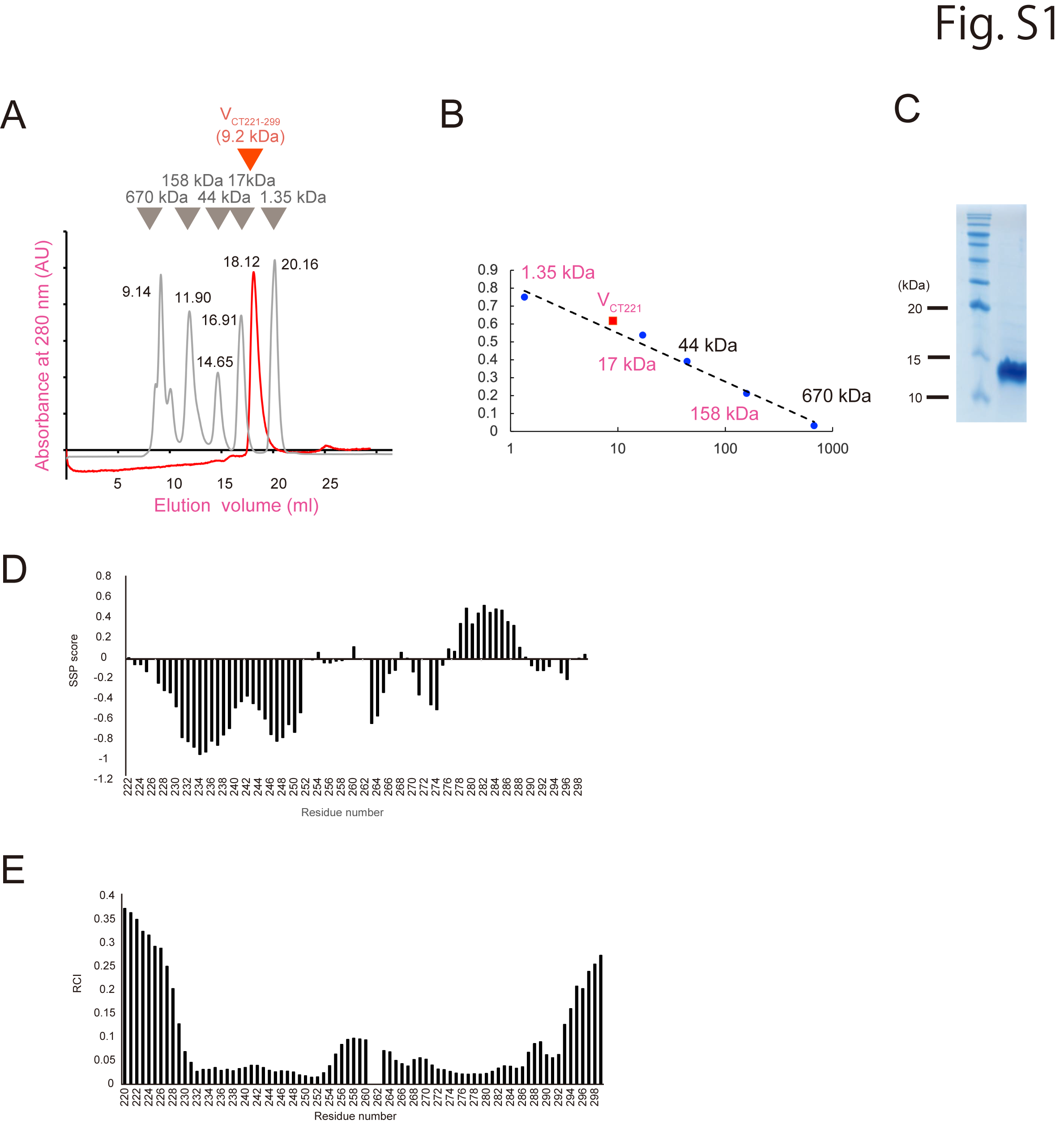

Supplement: Fig. S1 — Biochemical characterization of MeV-VCT221-299. [file jvi.00739-25-s0001.tif]
