# Supplementary material for: Solution structure of the C-terminal domain of the measles virus V protein in its free form and mechanistic analysis of STAT2 targeting
Source: J Virol. 2025 Sep 11;99(10):e00739-25. doi: 10.1128/jvi.00739-25 (PMC12548473; doi:10.1128/jvi.00739-25)
Supplement: Supplemental legends — Legends for Fig. S1 and Videos S1 to S3. [file jvi.00739-25-s0002.docx]

**Supplemental legends**

**Figure S1** Biochemical characterization of MeV-V_CT221-299_. (**A**) SEC profile of MeV-V_CT221-299_ (red trace), overlaid with standard protein markers: (i) 670 kDa (thyroglobulin), (ii) 158 kDa (γ-globulin), (iii) 44 kDa (ovalbumin), (iv) 17 kDa (myoglobin), and (v) 1.35 kDa (vitamin B_12_). The chromatogram of the protein standards is shown in gray. Retention volumes of the MeV-V_CT221-299_ and the protein standard proteins are indicated with their molecular weights. The vertical value is arbitrary unit (AU). **(B)** Calibration curve for molecular weight estimation, constructed by plotting the gel-phase distribution coefficient *K*av versus the logarithm of the molecular weight (log *M*_w_). *K*_av_ was calculated as *K*_av_ = (*V*_e_ − *V*_o_)/(*V*_c_ − *V*_o_), where *V*_e_ is the elution volume, *V*_o_ is the column void volume (8.64 ml), and *V*_c_ is the column bed volume (24.0 ml). The linear regression (blue circles) yielded the equation Y = −0.27175 × logX + 1.6351 (R² = 0.9842). Red square represents the *K*av value for MeV-V_CT221-299_, corresponding to an estimated molecular weight of 5.6 kDa. (**C**) SDS–PAGE analysis of purified MeV-V_CT221-299_, confirming sample purity. (**D**) Residue-specific Secondary Structure Propensity (SSP) scores for MeV-V_CT221-299_, calculated from backbone chemical shifts. **(E)** Residue-specific Random Coil Index (RCI) values of MeV-V_CT221-299_, reflecting local backbone flexibility.

**Video S1**

**A rotation movie along the y axis of Fig. 2B.**

**Video S2**

**A rotation movie along the y axis of Fig. 4B.**

**Video S3**

**A rotation movie along the y axis of Fig. 4D.**
